# LncRNA NORAD通过miR-199a-3p调控ZNF217对非小细胞肺癌细胞增殖、凋亡及化疗敏感性的影响

**DOI:** 10.3779/j.issn.1009-3419.2023.102.27

**Published:** 2023-07-20

**Authors:** Ying GAO, Xiaolin LUO, Pengfei LIAO, Yuanyuan LUO

**Affiliations:** ^1^425000 永州，永州职业技术学院护理学院; ^1^Nursing School, Yongzhou Vocational and Technical College; ^2^425000 永州，永州职业技术学院附属医院呼吸科; ^2^Department of Respiratory, Affiliated Hospital of Yongzhou Vocational and Technical College; ^3^425000 永州，永州职业技术学院医学院; ^3^Medical School, Yongzhou Vocational and Technical College, Yongzhou 425000, China

**Keywords:** LncRNA NORAD, miR-199a-3p, ZNF217, 肺肿瘤, 化疗敏感性, LncRNA NORAD, miR-199a-3p, ZNF217, Lung neoplasms, Chemotherapy sensitivity

## Abstract

**背景与目的** 针对非小细胞肺癌（non-small cell lung cancer, NSCLC）的治疗和诊断仍然是医学界的难题，而探究NSCLC发生发展的分子机制是当前研究的热点。长链非编码RNA（long non-coding RNA, lncRNA）NORAD在多种癌细胞中高表达，可能是促进NSCLC发生的分子靶点。探讨LncRNA NORAD通过miR-199a-3p调控锌指蛋白217（zinc finger protein 217, ZNF217）对NSCLC细胞增殖、凋亡及化疗敏感性的影响。**方法** 实时定量聚合酶链式反应（real-time quantitative polymerase chain reaction, qRT-PCR）法检测正常肺上皮细胞BEAS-2B、肺癌H460细胞和顺铂（Cisplatin, DDP）耐药细胞株H460/DDP细胞中NORAD、miR-199a-3p、ZNF217基因表达；将H460/DDP细胞分为control组、si-NC组、si-NORAD组、miR-NC组、miR-199a-3p mimic组、si-NORAD+inhibitor NC组、si-NORAD+miR-199a-3p inhibitor组。检测细胞增殖、细胞凋亡情况，各组细胞NORAD、miR-199a-3p、ZNF217基因表达，Ki-67、caspase-9、ZNF217蛋白表达量；验证miR-199a-3p与NORAD和ZNF217的关系。**结果** 与BEAS-2B细胞相比，H460和H460/DDP细胞中NORAD、ZNF217 mRNA表达显著升高（P<0.05），miR-199a-3p表达显著降低（P<0.05）。与H460细胞相比，H460/DDP细胞中NORAD、ZNF217 mRNA表达显著升高（P<0.05），miR-199a-3p表达显著降低（P<0.05）。与control组和si-NC组相比，si-NORAD组H460/DDP细胞增殖率、NORAD、ZNF217 mRNA表达、Ki-67、ZNF217蛋白表达显著降低（P<0.05），凋亡率、miR-199a-3p表达、caspase-9表达显著升高（P<0.05）。与miR-NC组相比，miR-199a-3p mimic组H460/DDP细胞增殖率、NORAD、ZNF217 mRNA表达、Ki-67、ZNF217蛋白表达显著降低（P<0.05），凋亡率、miR-199a-3p、caspase-9表达显著升高（P<0.05）。与si-NORAD+inhibitor NC组相比，si-NORAD+miR-199a-3p inhibitor组H460/DDP细胞增殖率、ZNF217 mRNA表达、Ki-67、ZNF217蛋白表达显著升高，凋亡率、miR-199a-3p表达、caspase-9表达显著降低（P<0.05）。**结论** 下调NORAD表达可增强miR-199a-3p表达并抑制ZNF217表达，进而抑制H460/DDP细胞增殖，促进其凋亡，增强其DDP化疗敏感性。

## Abstract (en)

**Background and objective** The treatment and diagnosis of non-small cell lung cancer (NSCLC) is still a difficult problem in the medical community, and exploring the molecular mechanism of the occurrence and development of NSCLC is a hot topic of the current research. Long non-coding RNA (lncRNA) NORAD is highly expressed in a variety of cancer cells. It may be a molecular target that promotes NSCLC. The aim of this study was to investigate the impacts of lncRNA NORAD on the proliferation, apoptosis, and chemosensitivity of NSCLC by regulating zinc finger protein 217 (ZNF217) through miR-199a-3p. **Methods** Real-time quantitative polymerase chain reaction (qRT-PCR) method was applied to detect the expressions of NORAD, miR-199a-3p and ZNF217 genes in normal lung epithelial cells BEAS-2B, lung cancer H460 cells, and Cisplatin (DDP) resistant cell lines H460/DDP. H460/DDP cells were devided into control group, si-NC group, si-NORAD group, miR-NC group, miR-199a-3p mimic group, si-NORAD+inhibitor NC group and si-NORAD+miR-199a-3p inhibitor group. Cell proliferation, apoptosis, the expression of NORAD, miR-199a-3p and ZNF217 genes of cells in each group were detected and the expression levels of Ki-67, caspase-9 and ZNF217 proteins in different cells were also observed. The relationship between miR-199a-3p, NORAD and ZNF217 was vefified. **Results** Compared with BEAS-2B cells, the expressions of NORAD, ZNF217 mRNA were significantly increased in H460 and H460/DDP cells (P<0.05) but the expression of miR-199a-3p was significantly reduced (P<0.05). Compared with H460 cells, the expression of NORAD and ZNF217 mRNA in H460/DDP cells was significantly increased (P<0.05) and the expression of miR-199a-3p was significantly reduced (P<0.05). Compared with the control group and si-NC group, the proliferation rate, NORAD and ZNF217 mRNA expression, Ki-67 and ZNF217 protein expression of H460/DDP cells in the si-NORAD group were significantly reduced (P<0.05), but the apoptosis rate, miR-199a-3p expression and caspase-9 expression were significantly increased (P<0.05). Compared with the miR-NC group, the proliferation rate, NORAD and ZNF217 mRNA expression, Ki-67 and ZNF217 protein expression of H460/DDP cells in the miR-199a-3p mimic group were significantly reduced (P<0.05), but the apoptosis rate, miR-199a-3p expression and caspase-9 expression were significantly increased (P<0.05). Compared with the si-NORAD+inhibitor NC group, the proliferation rate, ZNF217 mRNA expression, Ki-67 and ZNF217 protein expression of H460/DDP cells in the si-NORAD+miR-199a-3p inhibitor group were significantly increased (P<0.05), the apoptosis rate, miR-199a-3p expression and caspase-9 expression were obviously increased reduced (P<0.05). **Conclusion** Down-regulating NORAD expression can enhance miR-199a-3p expression and inhibit ZNF217 expression, thereby inhibiting H460/DDP cell proliferation, promoting apoptosis and enhancing its DDP chemotherapy sensitivity.

非小细胞肺癌（non-small cell lung cancer, NSCLC）是最常见的肺癌类型，在肺癌患者中占85%左右^[1]^。引起肺癌的因素有很多，如长期吸入粉尘、吸烟等，但具体发病机制尚不完全清楚。虽然针对肺癌的治疗手段发展迅速，但其预后差，治愈率低，死亡率高^[2]^。目前，化疗仍然是治疗癌症的常规手段，但在治疗过程中容易出现耐药性，导致治疗效果越来越差^[3]^，因此寻找提高肺癌对药物敏感性的机制成为治疗肺癌的研究方向。长链非编码RNA（long non-coding RNA, lncRNA）是调节基因转录和调控生物生长代谢过程的参与者，在肿瘤细胞生长过程中发挥重要作用^[4]^。李欢等^[5]^研究表明lncRNA NORAD在食管鳞状细胞EC9706中高表达，敲低lncRNA NORAD可抑制EC9706细胞活性。Wang等^[6]^研究表明lncRNA NORAD在NSCLC组织和细胞中上调，通过靶向miRNA-455/CDK14轴增强肿瘤细胞增殖能力，进而促进NSCLC的发生发展。miRNA是一类非编码小RNA，通过与靶基因的碱基互补结合来调控基因表达，参与肿瘤细胞生长过程^[7]^。Liu等^[8]^研究表明miR-199a-3p在NSCLC组织和细胞系中低表达，过表达miR-199a-3p可显著抑制体内肿瘤生长。Bai等^[9]^研究表明过表达miR-199a-3p可下调ZEB3表达进而抑制小鼠体内NSCLC细胞生长，并促进其凋亡。锌指蛋白217（zinc finger protein 217, ZNF217）属于锌指蛋白家族，在多种癌细胞中高表达并促进癌症的发生发展^[10]^。张惠丽等^[11]^研究表明ZNF217在NSCLC细胞中高表达，下调其表达可抑制NSCLC细胞增殖，并促进细胞凋亡。miR-199a-3p与NORAD和ZNF217存在靶向结合位点，但NORAD是否可以通过调节miR-199a-3p/ZNF217影响肺癌细胞药物敏感性尚不清楚。因此本研究就lncRNA NORAD通过miR-199a-3p调控ZNF217对NSCLC细胞增殖、凋亡及化疗敏感性的影响进行探究。

## 1 材料与方法

### 1.1 实验细胞

正常肺上皮细胞BEAS-2B、肺癌H460细胞和顺铂（Cisplatin, DDP）耐药细胞株H460/DDP均购自中国科学院上海细胞库。

### 1.2 试剂

DDP购自上海阿拉丁生化科技股份有限公司；RPMI-1640培养基购自河南鲲锦生物科技有限公司；CCK-8试剂盒购自南京森贝生物科技有限公司；胎牛血清购自厦门逸漠生物科技有限公司；Trizol试剂购自南京广兰生物科技有限公司；总RNA提取试剂盒购自福州奥研实验器材有限责任公司；实时定量聚合酶链式反应（real-time quantitative polymerase chain reaction, qRT-PCR）试剂盒购自上海赫澎生物科技有限公司；双荧光素酶报告基因检测试剂盒购自北京力波生物科技有限公司；Ki-67、caspase-9、ZNF217一抗体购自英国Abcam公司；si-NC、si-NORAD、miR-NC、miR-199a-3p mimic、inhibitor NC、miR-199a-3p inhibitor购自上海吉凯基因医学科技有限公司。

### 1.3 细胞培养

将BEAS-2B细胞、肺癌H460细胞和H460/DDP细胞分别接种到RPMI-1640培养基，耐药细胞培养另外添加0.5 μg/mL的DDP培养（37 ^o^C, 5%CO_2_）。

### 1.4 qRT-PCR检测BEAS-2B、H460和H460/DDP细胞中NORAD、miR-199a-3p、ZNF217 mRNA表达水平

用Trizol试剂提取各组细胞中的总RNA，反转录试剂盒将RNA逆转录为cDNA，荧光定量PCR扩增cDNA。NORAD（XM_054323966.1, 177 bp）、ZNF217 mRNA（NR_027451.1, 136 bp）以GAPDH为内参，miR-199a-3p（NC_000075.7, 21 bp）以U6为内参，使用2^-ΔΔCt^方法计算NORAD、miR-199a-3p、ZNF217 mRNA的相对表达量。引物序列：NORAD：F：5ʹ-CTATTTGTAAATACCTTTGTTATTAAT-3ʹ；R：5ʹ-ACATACAGTCCTGAACAAGTAATCCA-3ʹ；miR-199a-3p：F：5ʹ-GCACAGTAGTCTGCACATTGG-3ʹ；R：5ʹ-GTGCAGGGTCCGAGGTATTC-3ʹ；ZNF217 mRNA：F：5ʹ-AGTGAGCCACATCCCAAAAGA-3ʹ；R：5ʹ-ACACTGGGTGACTCCACCTC-3ʹ；GAPDH：F：5'-TGTAGACCATGTAGTTGAGGTCA-3'；R：5'-AGGTCGGTGTGAACGGATTTG-3'。U6：F：5'-CTCGCTTCGGCAGCACA-3'；R：5'-AACGCTTCACGAATTTGCGT-3'。

### 1.5 分组及处理

将对数期H460/DDP细胞分为control组、si-NC组（转染si-NC）、si-NORAD组（转染si-NORAD）、miR-NC组（转染miR-NC）、miR-199a-3p mimic组（转染miR-199a-3p mimic）、si-NORAD+inhibitor NC组（共转染si-NORAD和inhibitor NC）、si-NORAD+miR-199a-3p inhibitor组（共转染si-NORAD和miR-199a-3p inhibitor），各组加入2 μg/mL的DDP处理24 h，进行后续实验。

### 1.6 CCK-8法检测细胞增殖

将各组H460/DDP细胞接种于96孔板中，按照1.5中分组处理，培养48 h，每孔各加入10 μL CCK-8溶液，37 ^o^C孵育30 min，酶标仪测量各孔的吸光度（波长450 nm），计算细胞增殖率。

### 1.7 流式细胞术检测细胞凋亡

各组H460/DDP细胞以每孔1×10^6^个接种在96孔板中培养过夜，PBS缓冲液清洗，离心收集细胞，根据凋亡试剂盒说明书要求，加入Annexin V-FITC与PI试剂，反应后，上样用流式细胞仪检测细胞凋亡。

### 1.8 qRT-PCR检测各组H460/DDP细胞中NORAD、miR-199a-3p、ZNF217 mRNA表达水平

操作步骤同1.4。

### 1.9 Western blot检测相关蛋白表达水平

提取各组细胞总蛋白质，并检测蛋白浓度。SDS-PAGE凝胶电泳分离蛋白并转移到封闭液中封闭之后加入Ki-67、caspase-9、ZNF217一抗（稀释1:1500）4 ^o^C摇床过夜，洗膜后再加入二抗（稀释1:5000）孵育2 h，使用ECL发光液显影，用Image-Pro Plus进行定量分析蛋白表达。

### 1.10 双荧光素酶实验验证miR-199a-3p与NORAD和ZNF217的靶向关系

构建NORAD野生型载体（NORAD-WT）和突变型载体（NORAD-MUT），将NORAD-WT和NORAD-MUT分别与miR-NC和miR-199a-3p mimic共转染于H460/DDP细胞中，48 h后，检测荧光素酶活性。

构建ZNF217野生型载体（ZNF217-WT）和突变型载体（ZNF217-MUT）将ZNF217-WT和ZNF217-MUT分别与miR-NC和miR-199a-3p mimic共转染于H460/DDP细胞中，48 h后检测荧光素酶活性。

### 1.11 统计分析

采用SPSS 26.0软件分析数据，计量资料用均数±标准差（Mean±SD）表示，多组间比较采用单因素方差分析，组内两两相互比较采用SNK-q检验。P<0.05表示差异有统计学意义。

## 2 结果

### 2.1 NORAD、miR-199a-3p、ZNF217 mRNA在各种细胞中表达水平分析

与BEAS-2B细胞相比，H460和H460/DDP细胞中NORAD、ZNF217 mRNA表达显著升高，miR-199a-3p表达显著降低（P<0.05）；与H460细胞相比，H460/DDP细胞中NORAD、ZNF217 mRNA表达显著升高，miR-199a-3p表达显著降低（P<0.05），见表1。

**表1:** 各种细胞系中NORAD、miR-199a-3p、ZNF217 mRNA表达比较（Mean±SD, n=6）

Group	NORAD	miR-199a-3p	ZNF217 mRNA
BEAS-2B cells	1.02±0.07	0.95±0.08	1.08±0.11
H460 cells	1.74±0.08^a^	0.68±0.06^a^	1.57±0.13^a^
H460/DDP cells	2.18±0.19^ab^	0.34±0.03^ab^	1.94±0.16^ab^

^a^: Compared with BEAS-2B cells, P<0.05; ^b^: Compared with H460 cells, P<0.05.ZNF217: zinc finger protein 217.

### 2.2 抑制NORAD和miR-199a-3p表达对H460/DDP细胞增殖率的影响

si-NORAD组H460/DDP细胞增殖率显著低于control组和si-NC组（P<0.05）；miR-199a-3p mimic组H460/DDP细胞增殖率显著低于miR-NC组（P<0.05）；si-NORAD+miR-199a-3p inhibitor组H460/DDP细胞增殖率显著高于si-NORAD+inhibitor NC组（P<0.05），见表2。

**表2:** 各组H460/DDP细胞增殖率比较（Mean±SD, n=6）

Group	Proliferation rate (%)
Control	100.00
si-NC	96.54±6.93
si-NORAD	51.37±4.76^ab^
miR-NC	94.26±7.42
miR-199a-3p mimic	43.84±4.47^c^
si-NORAD+inhibitor NC	48.67±5.24
si-NORAD+miR-199a-3p inhibitor	77.58±6.92^d^

^a^: Compared with control, P<0.05; ^b^: Compared with si-NC, P<0.05; ^c^: Compared with miR-NC, P<0.05; ^d^: Compared with si-NORAD+inhibitor NC, P<0.05.

### 2.3 抑制NORAD和miR-199a-3p表达对H460/DDP细胞凋亡率的影响

si-NORAD组H460/DDP细胞凋亡率显著高于control组和si-NC组（P<0.05）；miR-199a-3p mimic组H460/DDP细胞凋亡率显著高于miR-NC组（P<0.05）；si-NORAD+miR-199a-3p inhibitor组H460/DDP细胞凋亡率显著低于si-NORAD+inhibitor NC组（P<0.05），见图1和表3。

**图1:**
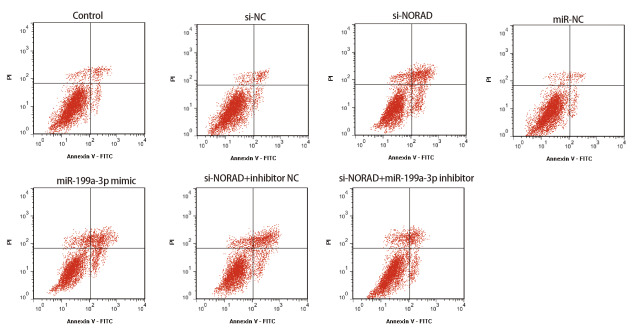
各组H460/DDP细胞凋亡率图

**表3:** 各组H460/DDP细胞凋亡率比较（Mean±SD, n=6）

Group	Apoptosis rate (%)
Control	6.57±0.82
si-NC	7.26±0.87
si-NORAD	36.48±3.62^ab^
miR-NC	6.85±0.93
miR-199a-3p mimic	39.72±3.86^c^
si-NORAD+inhibitor NC	34.68±3.43
si-NORAD+miR-199a-3p inhibitor	18.54±2.36^d^

^a^: Compared with control, P<0.05; ^b^: Compared with si-NC, P<0.05; ^c^: Compared with miR-NC, P<0.05; ^d^: Compared with si-NORAD+inhibitor NC, P<0.05.

### 2.4 抑制NORAD和miR-199a-3p表达对NORAD、miR-199a-3p、ZNF217 mRNA表达的影响

与control组和si-NC组相比，si-NORAD组H460/DDP细胞NORAD、ZNF217 mRNA表达显著降低，miR-199a-3p表达显著升高（P<0.05）；与miR-NC组相比，miR-199a-3p mimic组H460/DDP细胞NORAD、ZNF217 mRNA表达显著降低，miR-199a-3p表达显著升高（P<0.05）；与si-NORAD+inhibitor NC组相比，si-NORAD+miR-199a-3p inhibitor组H460/DDP细胞ZNF217 mRNA表达显著升高，miR-199a-3p mRNA表达显著降低（P<0.05），NORAD mRNA表达无统计学差异（P>0.05），见表4。

**表4:** 各组H460/DDP细胞中NORAD、miR-199a-3p、ZNF217 mRNA表达比较（Mean±SD, n=6）

Group	NORAD	miR-199a-3p	ZNF217 mRNA
Control	2.18±0.19	0.34±0.03	1.94±0.16
si-NC	2.11±0.17	0.36±0.04	1.98±0.17
si-NORAD	1.46±0.12^ab^	0.78±0.07^ab^	1.37±0.11^ab^
miR-NC	2.14±0.17	0.37±0.03	2.02±0.18
miR-199a-3p mimic	1.32±0.11^c^	0.84±0.08^c^	1.22±0.09^c^
si-NORAD+inhibitor NC	1.48±0.14	0.76±0.07	1.39±0.12
si-NORAD+miR-199a-3p inhibitor	1.51±0.13	0.58±0.06^d^	1.72±0.15^d^

^a^: Compared with control, P<0.05; ^b^: Compared with si-NC, P<0.05; ^c^: Compared with miR-NC, P<0.05; ^d^: Compared with si-NORAD+inhibitor NC, P<0.05.

### 2.5 抑制NORAD和miR-199a-3p表达对H460/DDP细胞中相关蛋白表达水平的影响

与control组和si-NC组相比，si-NORAD组H460/DDP细胞Ki-67、ZNF217表达显著降低，caspase-9表达显著升高（P<0.05）；与miR-NC组相比，miR-199a-3p mimic组H460/DDP细胞Ki-67、ZNF217表达显著降低，caspase-9表达显著升高（P<0.05）；与si-NORAD+inhibitor NC组相比，si-NORAD+miR-199a-3p inhibitor组H460/DDP细胞Ki-67、ZNF217表达显著升高，caspase-9表达显著降低（P<0.05），见图2和表5。

**图2:**
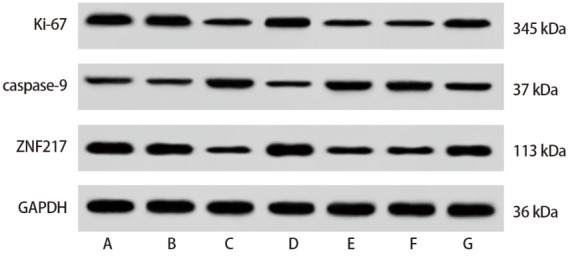
各组H460/DDP细胞中Ki-67、caspase-9、ZNF217蛋白表达

**表5:** 各组H460/DDP细胞中相关蛋白表达水平比较（Mean±SD, n=6）

Group	Ki-67	Caspase-9	ZNF217
Control	0.82±0.07	0.32±0.03	1.16±0.12
si-NC	0.78±0.06	0.35±0.04	1.09±0.10
si-NORAD	0.33±0.04^ab^	0.75±0.06^ab^	0.53±0.05^ab^
miR-NC	0.83±0.08	0.34±0.04	1.13±0.11
miR-199a-3p mimic	0.28±0.02^c^	0.83±0.08^c^	0.47±0.04^c^
si-NORAD+inhibitor NC	0.35±0.03	0.77±0.07	0.56±0.05
si-NORAD+miR-199a-3p inhibitor	0.61±0.05^d^	0.54±0.05^d^	0.95±0.08^d^

^a^: Compared with control, P<0.05; ^b^: Compared with si-NC, P<0.05; ^c^: Compared with miR-NC, P<0.05; ^d^: Compared with si-NORAD+inhibitor NC, P<0.05.

### 2.6 验证miR-199a-3p与NORAD和ZNF217的靶向关系

利用Starbase网站预测miR-199a-3p与NORAD和ZNF217结合位点，见图3。与miR-NC和NORAD-WT共转染组比较，miR-199a-3p mimic与NORAD-WT共转染组荧光素酶活性显著降低（P<0.05）；与NORAD-MUT和miR-NC组相比，NORAD-MUT和miR-199a-3p mimic共转染组光素酶活性差异无统计学意义（P>0.05），见图4。与miR-NC和ZNF217-WT共转染组比较，miR-199a-3p mimic和ZNF217-WT共转染组的荧光素酶活性显著降低（P<0.05）；与miR-NC和ZNF217-MUT共转染组比较，miR-199a-3p mimic和ZNF217-MUT共转染组荧光素酶活性变化无统计学差异（P>0.05），见图5。

**图3:**
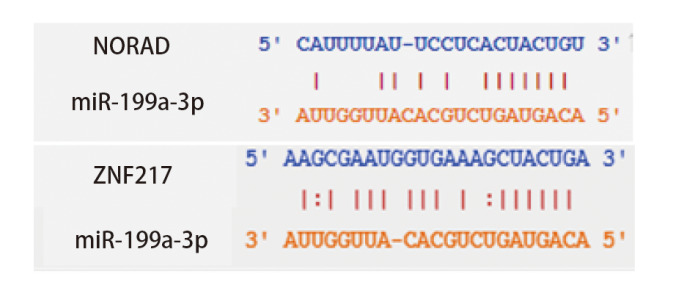
miR-199a-3p与NORAD和ZNF217结合位点

**图4:**
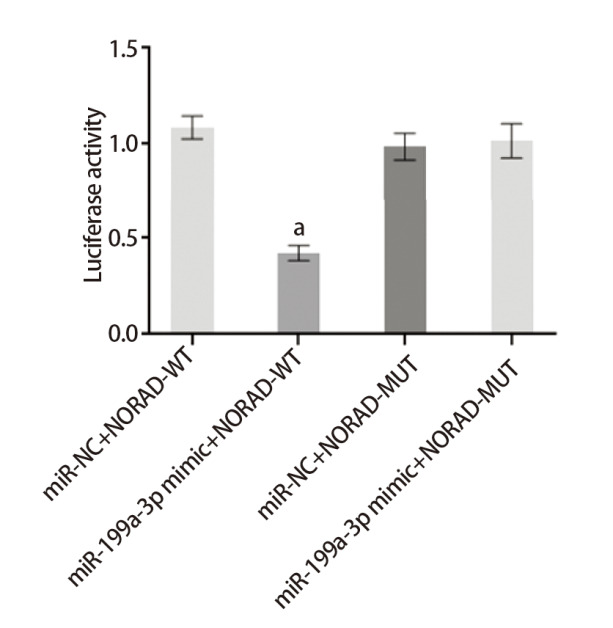
miR-199a-3p与NORAD的靶向关系验证

**图5:**
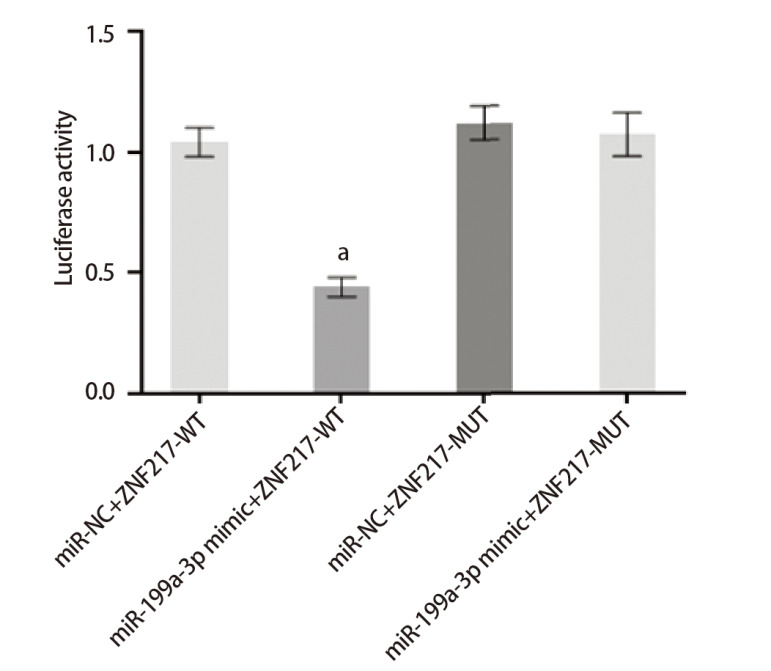
miR-199a-3p与ZNF217的靶向关系验证

## 3 讨论

肺癌的发病率和死亡率在所有癌症中居于首位，而以NSCLC患者最多^[12]^。个人生活习惯和所处外界环境因素是导致肺癌的主要因素，且随着年纪的增加肺癌发病率也随之升高^[13]^。目前针对NSCLC的治疗已经取得一定进展，但治疗效果仍不尽人意。晚期肺癌的治疗以化疗为主，但内源性和获得性化疗抵抗导致化疗效果大打折扣^[14]^，因此探究寻找提高肺癌细胞化疗敏感性的机制对治疗肺癌具有重要意义。

LncRNA是指长度超过200个核苷酸但没有蛋白质编码能力的RNA转录产物，但其可以在转录水平上调控基因表达^[15]^，其在癌症发生发展过程中发挥重要调节作用，还具有调节化疗耐药性的作用。郭盛虎^[16]^研究表明lncRNA TUG1可以抑制NSCLC细胞增殖、迁移，提高其对DDP的敏感性，促进其自噬和凋亡。李正雄等^[17]^研究表明FTH1P3通过下调miR-218，可以降低A549/DDP细胞对DDP的敏感性，敲低FTH1P3可以提高A549/DDP细胞对DDP的敏感性。Caspase-9是线粒体凋亡通路的重要启动子和关键蛋白酶，具有启动凋亡和调节效应型caspase的活性。本研究结果表明，NORAD在H460和H460/DDP细胞中高表达，推测NORAD可能与H460细胞耐药性有关。下调NORAD表达后发现，H460/DDP细胞增殖率、Ki-67蛋白表达显著降低，凋亡率、caspase-9表达显著升高。提示干扰NORAD后可以抑制H460/DDP细胞增殖，促进其凋亡，增强其对DDP的敏感性。

有研究^[18]^表明，miRNA在转录或转录后可调控基因表达，进而参与细胞的生物学过程。Dong等^[19]^研究表明过表达miR-199-3p可抑制CHML与Rab5A结合进而抑制NSCLC细胞的增殖并促进其凋亡。熊伟杰等^[20]^研究表明过表达miR-199a-3p可抑制肺癌A549细胞恶性生物学行为。本研究结果表明，miR-199a-3p在H460和H460/DDP细胞中低表达，上调miR-199a-3p表达后，H460/DDP细胞增殖率、Ki-67表达显著降低，凋亡率、caspase-9表达显著升高。提示过表达miR-199a-3p也可以抑制H460/DDP细胞增殖，促进其凋亡，增强其对DDP敏感性。

研究^[21,22]^证实，lncRNA与miRNA存在结合位点，可以调控miRNA表达，进而影响细胞生物学进程。Huang等^[21]^研究表明lncRNA NORAD通过靶向miR-129-1-3p/SOX4轴可以增强肺癌A549/DDP和H446/DDP细胞对DDP的耐药性，促进细胞增殖，敲低NORAD可增强其对DDP的敏感性，抑制细胞增殖。Shen等^[22]^研究表明NORAD通过海绵化miR-549-1-202p可以增强A549/DDP细胞对DDP的耐药性，促进细胞增殖。本研究通过生物信息学显示miR-199a-3p与NORAD存在结合位点，敲除NORAD后miR-199a-3p表达显著升高，ZNF217表达显著降低，同时可以抑制H446/DDP细胞增殖。在敲除NORAD的基础上，下调miR-199a-3p表达，发现下调miR-199a-3p可以部分逆转敲除NORAD对A549/DDP细胞增殖的抑制作用。双荧光素酶报告实验也证明两者的靶向关系，证实miR-199a-3p是NORAD的靶标。提示NORAD可以调控miR-199a-3p表达进而影响A549/DDP细胞的增殖、凋亡和化疗敏感性。为验证miR-199a-3p和ZNF217的关系，本研究通过过表达miR-199a-3p，发现ZNF217表达显著降低，A549/DDP细胞增殖率显著降低，凋亡率显著升高。提示miR-199a-3p可下调ZNF217的表达，进而抑制H460/DDP细胞增殖，促进其凋亡，增强其对DDP的敏感性。

综上所述，干扰NORAD可以上调miR-199a-3p表达，下调ZNF217表达，抑制H460/DDP细胞增殖，促进其凋亡，增强其DDP化疗敏感性。本实验仅在细胞水平进行探究，后续还需进行动物实验在体内水平进行进一步验证。

**Competing interests**

The authors declare that they have no competing interests.

**Auhtors contributions**

Gao Y and Luo YY conceived and designed the study. Gao Y, Luo XL, Liao PF performed the experiments. Gao Y analyzed the data. Luo XL and Liao PF contributed analysis tools. Luo YY and Gao Y provided critical inputs on design, analysis and interpretation of the study. All the authors had access to the data. All authors read and approved the final manuscript as submitted.
